# Synchronous Bilateral Testicular Tumors With Discordant Histopathology: A Rare Case in an Infertile Male

**DOI:** 10.7759/cureus.49874

**Published:** 2023-12-03

**Authors:** Saif Dalla Ali, Ibrahim A Khalil, Bara Wazwaz, Nagy Younes, Khalid Al Rumaihi

**Affiliations:** 1 Department of Education, Hamad Medical Corporation, Doha, QAT; 2 Department of Urology, Hamad Medical Corporation, Doha, QAT; 3 Department of Pathology, Hamad Medical Corporation, Doha, QAT

**Keywords:** discordant histopathology, embryonal carcinoma, seminoma, bilateral testicular cancer, testicular germ cell tumor

## Abstract

Testicular cancer, accounting for 1-1.5% of male malignancies, rarely presents bilaterally, with only 2-3% of cases being bilateral and a mere 10% being synchronous, typically sharing histological patterns in both testes. Discordant histological presentation is exceedingly rare, with only a few reported cases. In this report, we detail a case involving a 35-year-old infertile male with bilateral synchronous testicular tumors, each exhibiting different histopathologies. This case highlights the diagnostic intricacies and the necessity for tailored management in the face of such uncommon presentations. The implications of oncological treatment and fertility preservation significantly affect the patient's overall quality of life.

## Introduction

Testicular cancer, a rare malignancy, represents approximately 1-1.5% of all male-related malignancies. Despite its infrequency, it stands out as the most prevalent cancer affecting males in the age range of 15-44 years [1]. While around 2-3% of testicular tumors manifest bilaterally, the majority of these occurrences are metachronous. However, synchronous bilateral testicular tumors, constituting just 10% of bilateral cases, are characterized by shared histological patterns, with bilateral seminoma being the most commonly observed presentation [2,3]. Though exceedingly rare, discordant histological presentations have significant implications for treatment and follow-up plans. In this context, we present a rare case of bilateral synchronous testicular tumor with different histopathologies in a male with a history of infertility, shedding light on the challenges and complexities of managing such unique clinical scenarios.

## Case presentation

A 35-year-old male patient presented with left testicular swelling that had persisted for 2 months. Physical examination revealed a hard, mobile mass in the left testis, along with an atrophied right testis. The patient's history included infertility, but he successfully conceived via in-vitro fertilization six years prior to the current presentation. He also underwent sleeve gastrectomy one year before this presentation. Otherwise, his medical history is unremarkable. Given the newly discovered testicular swelling, an urgent ultrasound and tumor marker tests were ordered. The patient's lab tests showed elevated tumor markers and low testosterone levels with high gonadotropins, leading to a diagnosis of primary hypogonadism (Table 1).

**Table 1:** Laboratory test results and reference ranges. LDH: Lactate dehydrogenase; AFP: Alpha fetoprotein; BhCG: Beta human chorionic gonadotropin; FSH: Follicle-stimulating hormone; ALT: Alanine aminotransferase; AST: Aspartate aminotransferase; Alk Phos: Alkaline phosphatase.

Test (Units)	Results	Normal reference Range
LDH (U/L)	1212	135-225
AFP IU/mL	38	0-6
BhCG (mIU/mL)	19	0-2
FSH (IU/L)	47	1.5-12.4
LH (IU/L)	40.5	1.7-8.6
Testosterone (nmol/L)	4.8	10.40-37.44
Albumin (gm/L)	39	35-50
ALT(U/L)	20	0-41
AST (U/L)	18	0-40
Alk Phos (U/L)	79	60-80

**Figure 1:**
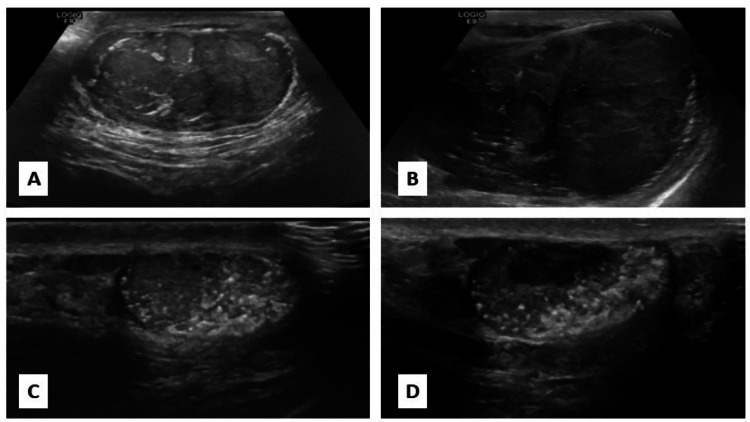
Ultrasound images showing development of new bilateral testicular masses. Ultrasound of the left testis showing the development of a new heterogeneous mass (B) that was not evident in the previous ultrasound (A). Ultrasound of the right testis showing a small testis (C) with the development of a small heterogeneous mass in the new ultrasound, without any change in testis size (D).

The patient’s record included a previous ultrasound of both testes. Starting with the left testis, there was a significant change from the previous size of 6 x 4 x 3.8 cm (49 mL) (Figure 1A) to 8.4 x 5.4 x 5.4 cm (127 mL). This was accompanied by diffuse heterogeneity and nodularity with calcifications and increased vascularity (Figure 1B), all consistent with a left testicular tumor. Regarding the right testis, the size remained unchanged from the previous ultrasound. However, there were diffuse parenchymal heterogeneity and calcifications, along with increased vascularity. Notably, a newly formed irregular hypoechoic area measuring 15mm, not present in the previous ultrasound, raised suspicion for another testicular tumor (Figures 1C-1D).

**Figure 2:**
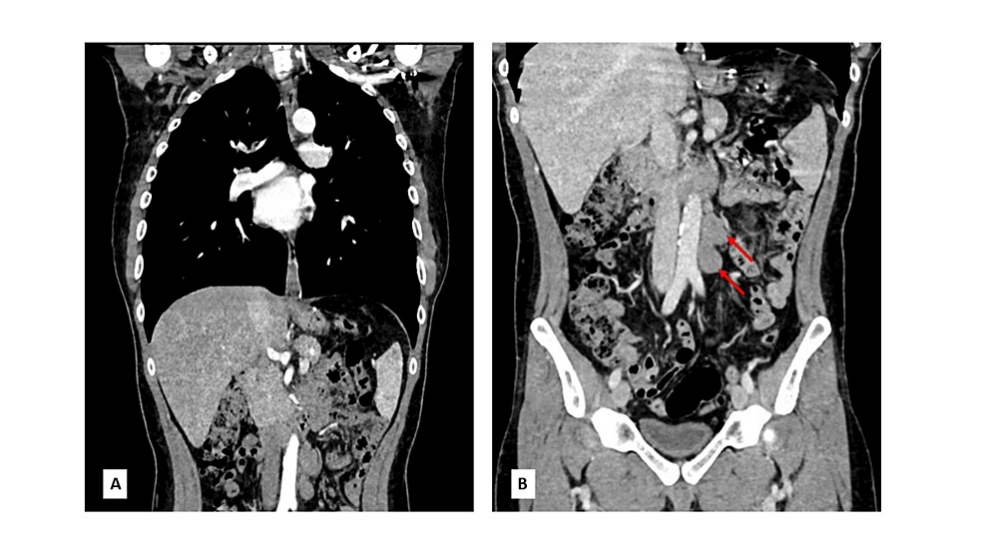
Staging chest, abdomen, and pelvis CT scan. A: Unremarkable CT of the chest with no metastatic lesions. B: CT of the abdomen showing two enlarged lymph nodes in the left para-aortic region (indicated by red arrows).

Staging chest, abdominal, and pelvic computed tomography (CT) scans revealed two enlarged lymph nodes in the left para-aortic region at the level of the left renal hilum, with a maximum diameter of 30 mm. There was no other evidence of metastatic disease (Figure 2).

**Figure 3:**
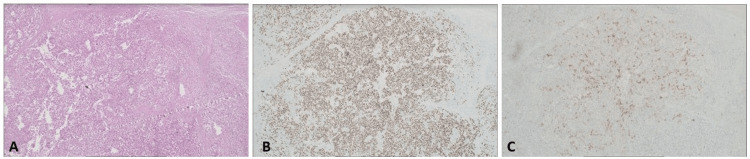
A: H&E stain showing sheets of seminoma tumor with focal polygonal cells representing embryonal carcinoma. B: Nuclear staining positive for Spalt-like transcription factor 4 (SALL4). C: Immunohistochemistry: CD30 highlighting the embryonal carcinoma component.

Following the aforementioned findings, the patient underwent bilateral radical orchiectomy with the insertion of bilateral testicular prostheses and sperm extraction. Histopathological diagnosis: Grossly, the cut surface of the left testicle revealed a 7.2 x 5.2 x 4.8 cm tan-yellow homogenous nodular soft mass replacing the entire testicle, while the right testicle exhibited a tan-white, smooth, rubbery, infiltrative mass measuring 1.9 x 1.4 x 0.8 cm, abutting the tunica albuginea. Microscopically, the mass in the left testicle was mainly composed of sheets of seminoma tumor cells with focal polygonal cells representing an embryonal carcinoma component (Figure 3A). Both components were positive for Sal-like transcription factor 4 (SALL4) (Figure 3B), while CD30 highlighted the embryonal carcinoma component (Figure 3C). Conversely, the right testicular mass showed a pure seminoma tumor. These findings correlate with a pathological stage of T3 in the left testicle and T1 in the right.

**Figure 4:**
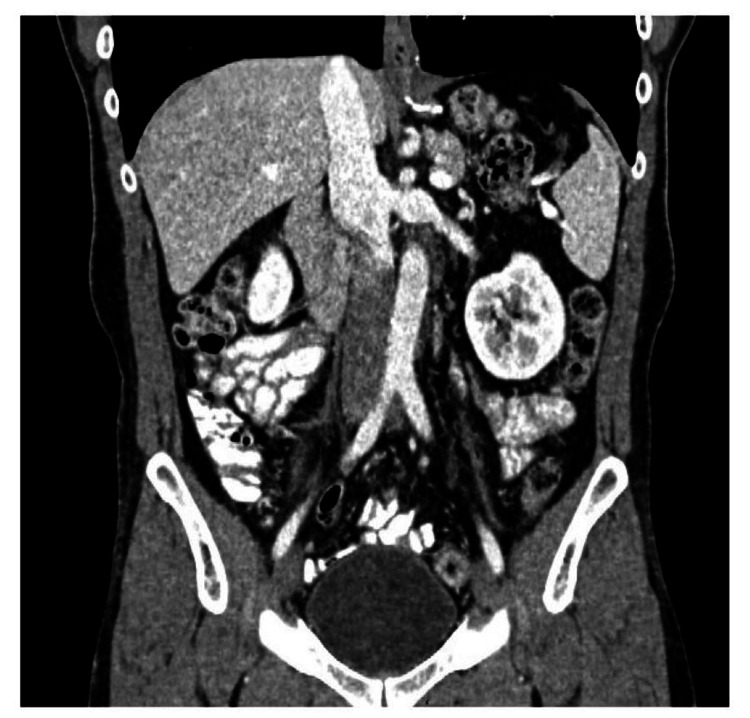
The CT scan of the abdomen showed resolution of the lymph nodes following adjuvant chemotherapy.

In order to plan adjuvant therapy for the patient, the highest stage was determined based on the pathology, tumor markers, and radiological findings. He was classified as having a pT3cN2M0 (STAGE IIb) tumor. Accordingly, he received adjuvant chemotherapy, which consisted of 4 cycles of Etoposide plus Cisplatin. Upon follow-up, the tumor markers normalized, and a CT scan showed complete resolution of the left paraaortic lymph nodes (Figure 4).

## Discussion

The WHO delineated testicular germ cell tumors in 2016 into two primary categories: prepubertal-type tumors, unrelated to germ cell neoplasia in situ (GCNIS), and postpubertal-type tumors, derived from GCNIS, commonly found in young men. The latter group comprises seminomas and non-seminomatous germ cell tumors, including embryonal carcinoma, yolk sac tumor, teratoma, and choriocarcinoma [4]. In our case study, an infertile adult presented with synchronous bilateral testicular tumors of distinct histopathologies. Upon examination, the right testis contained a pure seminoma, while the left testis harbored a mixed germ cell tumor.

Historically, the pathogenesis behind the development of synchronous bilateral testicular germ cell tumors (TGCTs) has been a controversial and yet-to-be-determined topic. Initially, when this subject was debated, researchers were divided over whether bilateral cancers were caused by metastasis or if separate oncologic mechanisms occurred concurrently. The latter possibility gained acceptance following a proposition by Matsushima M et al. in 1987. They argued that synchronous bilateral TGCTs resulted from separate oncologic mechanisms, citing the lack of vascular or lymphatic connections between the testes as evidence [5]. This proposition finds support in the varied activation, de-repression, and repression of distinct genes during the development of tumors with diverse histological subtypes [6]. As a result, it has been accepted that bilateral synchronous tumors of different histology follow a similar mechanism and occur as the development of two independent primary tumors.

A systematic review was conducted to assess the clinicopathological characteristics of bilateral TGCTs. It was found that synchronous bilateral TGCTs were associated with more advanced disease and a worse prognosis compared to metachronous bilateral TGCTs. The five-year survival rate for synchronous bilateral TGCTs was 88%, while it was 95% for metachronous bilateral TGCTs. Additionally, discordant histology was found to negatively impact the overall survival of the group with synchronous tumors [1].

Although it presents many complications, such as erectile dysfunction, infertility, and psychological distress, bilateral radical orchiectomy remains the standard of care for patients presenting with synchronous testicular cancer. Over the past decade, there has been increased focus on research into the treatment of bilateral testicular cancer. The aim is to identify approaches that preserve fertility and eliminate the need for lifelong androgen treatment, thereby significantly enhancing a patient's quality of life. Extensive research has also explored the viability of testicular-sparing surgery as an alternative to radical orchiectomy. Nonetheless, radical orchiectomy continues to be the standard of care, pending further advancements in understanding. In our case, the patient underwent bilateral radical orchiectomy, followed by adjuvant chemotherapy.

## Conclusions

It is known that bilateral primary synchronous testicular germ cell tumors (TGCTs) typically present with similar histology; however, it is rare for them to present with different histopathologies. Our patient presented with a case of primary synchronous bilateral testicular cancer, exhibiting discordant histology in each testis. The treatment of this condition should be based on the tumor with the highest stage and most malignant features.
